# Fat Mass and Obesity Associated Gene Polymorphism and the Risk of Polycystic Ovary Syndrome: A Meta-analysis

**Published:** 2017-01

**Authors:** Ying LIU, Yongxia CHEN

**Affiliations:** Dept. of Gynecology, the Second Affiliated Hospital of Guangzhou Medical University, Guangzhou, China

**Keywords:** FTO gene, SNP, PCOS, Meta-analysis

## Abstract

**Background:**

We aimed to elucidate the association between fat mass and obesity associated gene (FTO) polymorphism and the risk of polycystic ovary syndrome (PCOS) by meta-analysis.

**Methods:**

We searched PubMed and Embase databases to find the relevant studies. Odds ratios (ORs) and their corresponding 95% confidence intervals (CIs) were used for pooled analysis. Statistical analyses were carried out by using R 3.12 software. Heterogeneity was assessed using *I^ 2^* and *Q* statistics. *I^ 2 ^*>50% or *P*<0.05 was considered as heterogeneity statistically, and random effects model was used for pooled analysis. Otherwise, fixed-effect model was used.

**Results:**

Twelve eligible studies that published from 2008 to 2015 were included in this meta-analysis. The pooled analyses showed that rs9939609 polymorphism of FTO gene was significantly associated with risk of PCOS under A vs. T, AT vs. TT, AA vs. TT, AA vs. AT+TT and AA+AT vs. TT genetic models. However, for rs8050136 and rs1421085, significant association was only found under recessive genetic model.

**Conclusion:**

rs9939609 variation of FTO gene is significantly associated with risk of PCOS. However, the association between rs8050136, rs1421085, and PCOS is still unclear and needs further confirmation.

## Introduction

Polycystic ovary syndrome (PCOS) is a common endocrine disease among women of reproductive age (1–3). The typical symptoms of PCOS include infertility, obesity, and hairiness, caused by elevated male hormone. PCOS can also arouse anxiety and depression of the patients (4).

Up to now, the treatment of PCOS is still a challenge for physicians and medical researchers. The treatments are mainly adopted depending on the symptoms of the PCOS patients. For examples, letrozole and clomiphene are used to treat the infertility of PCOS (5). Some insulin-sensitizing drugs such as metformin, usually used to manage type 2 diabetes mellitus, are also applied to treat PCOS now (6). However, individual variations of the PCOS should be considered. For instance, a special PCOS patient may have normal male hormone levels. In addition, PCOS is associated with hyperinsulinemia and peripheral insulin resistance, and obesity can aggravate both abnormalities (7).

Because of the difficult treatment of PCOS, the medical researchers have tried to consider the risk factors associated with PCOS to prevent prevalence of the disease. Genetic and environmental factors have been identified to be associated with PCOS. The fat mass and obesity-associated gene (FTO) as an obesity candidate gene has been related to PCOS susceptibility (8). However, the results of the studies were confused. For example, a previous study (9) states that FTO is significantly associated with the risk of PCOS, but another study (10) reports no association between the two entities.

In this study we systematically meta-analyzed the genotypes of FTO gene and risk of PCOS, thus, to identify key variations of FTO gene associated with risk of PCOS.

## Materials and Methods

### Source of data

We searched two English databases PubMed and Embase using the search terms of (PCOS OR polycystic ovarian syndrome OR Polycystic ovary syndrome) and (FTO OR fat mass and obesity associated gene OR s1121980 OR rs1421085 OR rs1558902 OR rs8050136 or rs17817449 or rs9939609 or rs9930506). The deadline of the search was Mar 2016. Web of science and Google scholar were also used for checking leakage.

### Inclusion and exclusion criteria

Inclusion criteria included: 1) the study reported the association between FTO and FCOS; 2) the study provided the data for calculating the distribution of FTO in PCOS patients and non-PCOS population. The reviews, reports, comments and letters were excluded from this analysis.

### Data extraction and quality assessment

The following data extracted from included studies: name of the first author, publication year, location of the study, the number of PCOS patients (cases) and non-PCOS population (controls), age of the participants and Body Mass Index (BMI). The number of each genotype in cases and controls was also extracted.

Quality assessment was conducted using Newcastle-Ottawa Scale (NOS) (11) independently. The disagreements in the processes of data extraction and quality assessment were resolved by discussing with Yongxia Chen.

### Statistical analysis

Hardy-Weinberg equilibrium (HWE) test was performed using chi-square (12). Pooled analysis was carried out using R 3.12 software with the effect size of odds ratios (ORs) and their corresponding 95% confidence interval (CI). Heterogeneity was assessed using *I^2^* and *Q* statistics (13). *I^2^*>50% or *P*<0.05 was considered as heterogeneity statistically, and random effects model was used for pooled analysis. Otherwise, a fixed-effect model was used. Publication bias was assessed by using Egger’s test and *P*<0.05 represented a significant publication bias (14).

## Results

### Study selection

**Fig. 1:**
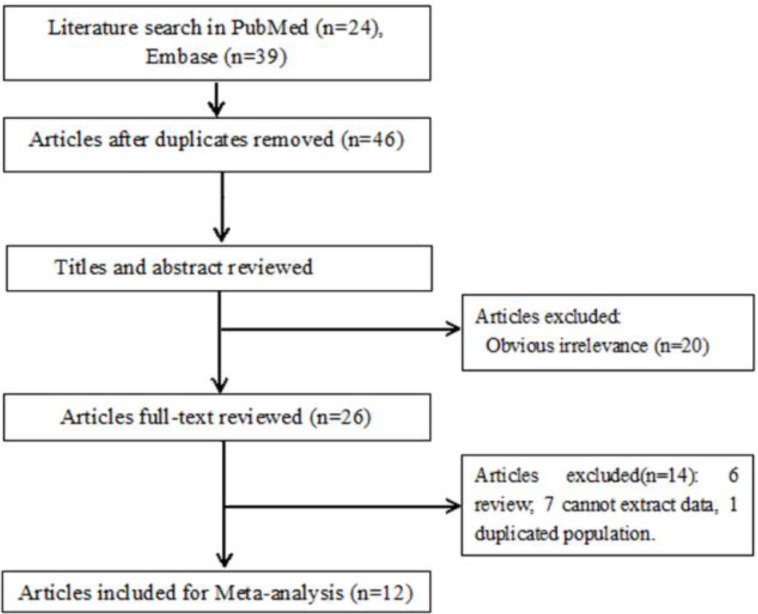
Literature search and study selection of this meta-analysis

The process of study selection was shown in Fig. 1.

Totally, 63 studies were identified by searching PubMed and Embase, and no additional studies were found from Web of science and Google scholar. Firstly, 17 duplicate studies were removed. Secondly, 19 irrelevant studies were excluded after reading titles and abstracts. Thirdly, 14 studies were removed from remaining 26 studies after reviewing full-texts. Finally, 12 eligible studies (8–10, 15–23) were included in this meta-analysis.

### Characteristics of included studies

The characteristics of 12 case-control studies were shown in Table 1. The publication year of included studies ranged from 2008 to 2015. These studies distributed in Korea, America, Brazil, and China. The major genotypes of FTO gene were rs17817449, rs1121980, rs1558902, rs11642841, rs17817449, rs9939609, rs8050136 and rs1421085. The quality assessment showed relative high quality with 6–8 scores of these included studies. HWE analysis (Table 2) showed that all the controls at rs9939609 loci conformed to HWE. However, two populations (17, 22) at rs8050136 loci and one population (15) at rs1421085 deviated from HWE (*P*<0.05).

**Table 1:** The characteristics of the included studies

**Author**	**Public year**	**Study location**	** P**	** C**	** P**	** C**	** P**	** C**	**Gene**	**NOS scores**
			**N**		**Age (y)**		**BMI (kg/m^2^)**			
Kim JJ et al. ( 10 )	2014	Korea	552	559	27.8±5.4	27.9±5.3	22.0±4.1	20.1±2.5	rs9939609	7
Attaoua R et al. ( 15 )	2008	France, Romania	207	100	24.3±0.6	34.1±1.1	27.4±0.7	22.2±0.4	rs1421085	6
Saxena R et al. ( 21 )	2013	USA	525	472	18–45				rs9939609, rs11642841	7
Ramos RB et al. ( 20 )	2015	Brazil	199	99	22.7±7.1		29.6± 6.4	27.0 ± 6.0	rs9939609, rs8050136	8
Song DK et al. ( 8 )	2014	Korea	432	927	24±5	27±5	24.0±4.7	21.1±2.6	rs1421085, rs8050136, rs17817449	6
Xue H et al. ( 22 )	2015	China	212	198	28.2±4.72	36.1±5.33	27.51±3.75	22.32±3.64	rs1121980, rs1421085, rs1558902, rs8050136	7
Yuan H et al. ( 23 )	2015	China	733	892	26.14±3.2 3	29.38±4.53	25.16±5.27	22.73±2.97	rs9939609	7
Barber T et al. ( 16 )	2008	UK	464	1336	32.3±7.0	41.5±11.45	27.5(21.2–35.7)	21.3–30.0	rs9939609	6
Hatziagelaki E et al. ( 17 )	2012	Germany	62	105	26±5.9	27±5.9	27.3±6.9	26.6±6.4	rs8050136	7
Kim JJ et al. ( 18 )	2012	Korea	698	386	28.1±5.2	28.5±4.9	21.9±3.4	20.1±2.3	rs1421085	7
Li T et al. ( 19 )	2013	China	3599	3082	28.35±3.75	31.33±4.69	24.81±4.29	22.73±3.15	rs9939609	6
Yan Q et al. ( 9 )	2009	China	215	227	21.7±5.5	27.5±4.8	28.0±6.1	20.8±3.3	rs9939609	6

P: polycystic ovary syndrome; C: Control; NOS: Newcastle-Ottawa Scale; N: The total number of including.

**Table 2:** The distribution of the FTO polymorphisms

**Gene**	**Author**	**Public year**	**N**	**PCOS**		**AA**	**N**	**Control**		**AA**	**HWE**	
				** TT**	** TA**			** TT**	** TA**		χ^2 ^^*^	***P***
rs9939609	Kim JJ et al. ( 10 )	2014	552	445	106	8	559	427	118	7	0.134	0.7146
	Yuan H et al. ( 23 )	2015	733	564	153	16	892	717	168	7	0.748	0.3871
	Barber T et al. ( 16 )	2008	464	133	231	99	1336	480	644	212	0.027	0.8696
	Saxena R et al. ( 21 )	2013	510	220		290	448	177		271	-	-
	Ramos RB et al. ( 20 )	2015	199	65	91	43	99	33	49	17	0.027	0.8700
	Li T et al. ( 19 )	2013	3599	2665	867	67	3082	2490	563	29	0.210	0.6468
	Yan Q et al. ( 9 )	2009	215	155	55	5	227	183	43	1	1.011	0.3147
			N	AA	AC	CC	N	AA	AC	CC		
rs8050136	Song DK et al. ( 8 )	2014	432	12	86	334	927	10	207	710	1.534	0.2155
	Xue H et al. ( 22 )	2015	212	6	44	162	198	6	32	160	5.124	0.0236
	Ramos RB et al. ( 20 )	2015	199	43	86	70	99	15	48	36	0.023	0.8783
	Hatziagelaki E et al. ( 17 )	2012	62	0	49	13	105	0	79	26	38.186	0.0000
			N	CC	CT	TT	N	CC	CT	TT		
rs1421085	Song DK et al. ( 8 )	2014	432	12	87	333	927	10	207	710	1.534	0.2155
	Xue H et al. ( 22 )	2015	212	5	42	165	198	2	39	157	0.063	0.8022
	Attaoua R et al. ( 15 )	2008	207	52	101	54	100	16	60	24	4.357	0.0369
	Kim JJ et al. ( 18 )	2012	601	17	158	426	386	12	93	281	1.436	0.2308

*: likelihood-ratio χ^2^; PCOS: polycystic ovary syndrome; HWE: Hardy-Weinberg equilibrium; Bold values mean to deviate from HWE

### Pooled analysis

**Fig. 2:**
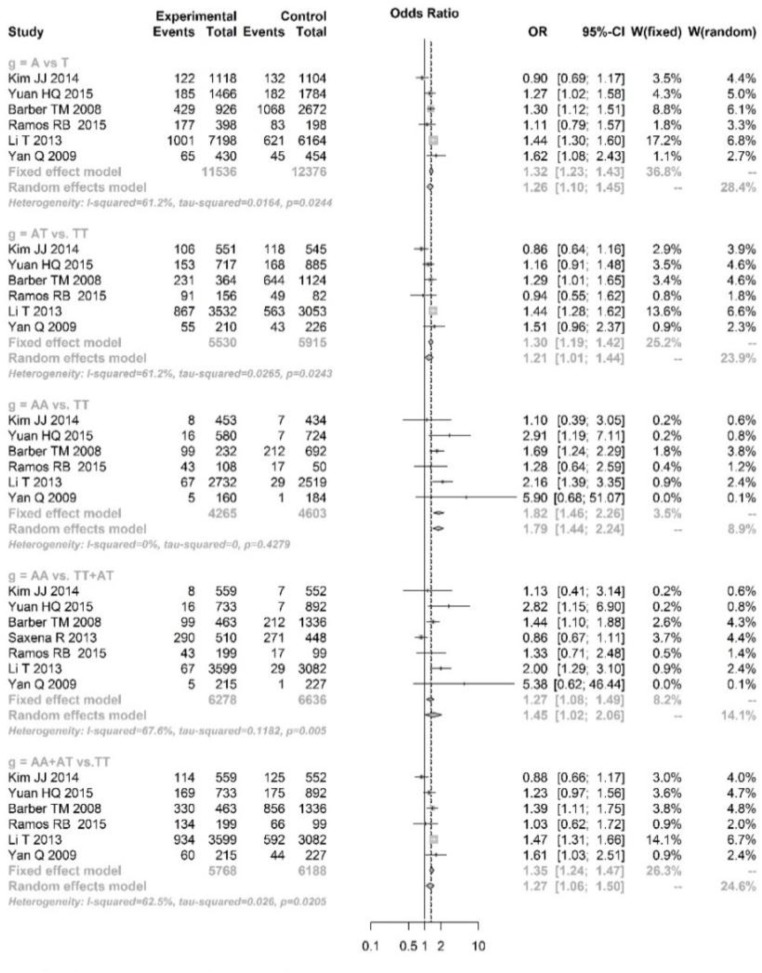
Pooled analysis of association between rs9939609 variation and polycystic ovary syndrome under allele (A vs T), additive (AT vs TT, AA vs TT), recessive (AA vs AT+TT) and dominant (AA+AT vs TT) genetic models

We meta-analyzed the association between PCOS and FTO gene polymorphisms at loci of rs9939609 (A vs T), rs8050136 (A vs C) and rs1421085 (C vs T) under allele, additive, recessive and dominant genetic models. As shown in Fig. 2, heterogeneity test showed significant heterogeneity in rs9939609 under allele (A vs. T, *I^2^*=61.2%), additive (AT vs. TT, *I^2^*=61.2%), recessive (AA vs. AT+TT, *I^2^*=67.6%) and dominant genetic models (AA+AT vs. TT, *I^2^*=62.5%). Therefore, the random effects model was used for pooling analysis. The analyses under other genetic models were pooled using fixed effect model.

**Fig. 3:**
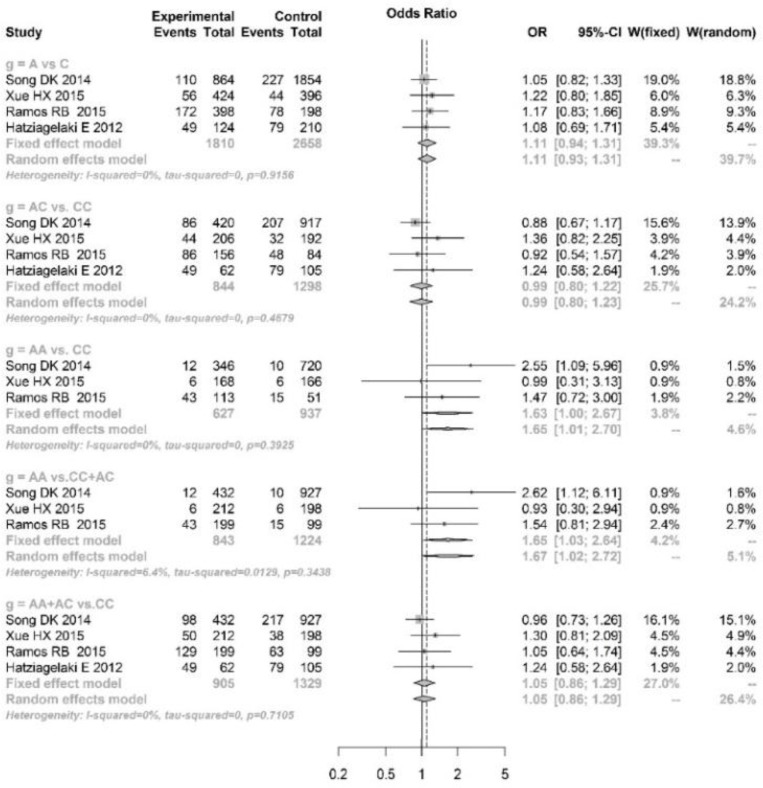
Pooled analysis of association between rs8050136 variation and polycystic ovary syndrome under allele (A vs C), additive (AA vs CC, AC vs CC), recessive (AA vs AC+CC) and dominant (AA+AC vs CC) genetic models

**Fig. 4:**
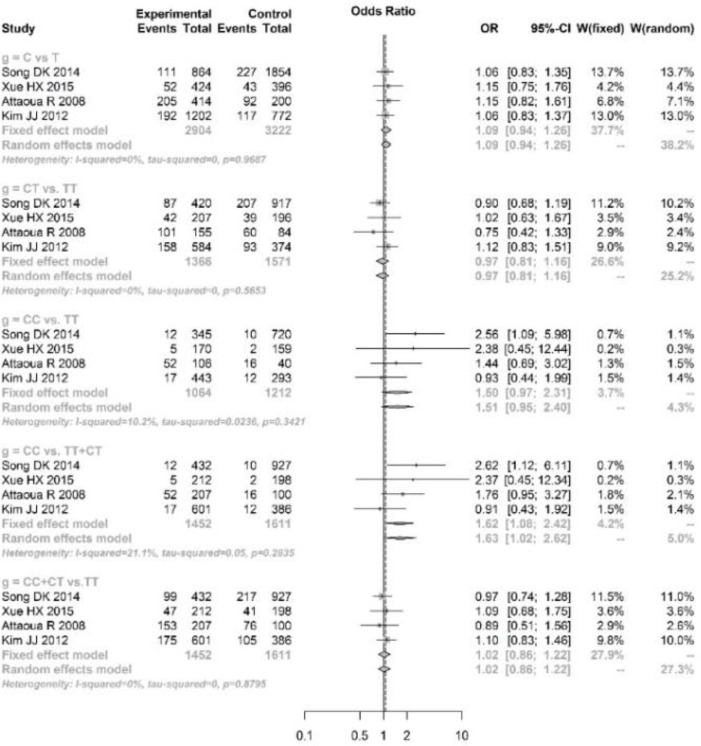
Pooled analysis of association between rs1421085 variation and polycystic ovary syndrome under allele (C vs T), additive (CT vs TT, CC vs TT), recessive (CC vs CT+TT) and dominant (CC+CT vs TT) genetic models

The pooled analyses showed that single nucleotide polymorphism (SNP) at rs9939609 of FTO gene was significantly associated with risk of PCOS under A vs. T (OR=1.26, 95%CI: 1.10–1.45), AT vs. TT (OR=1.21, 95%CI: 1.01–1.44), AA vs. TT (OR=1.82, 95%CI: 1.46–2.26), AA vs. AT+TT (OR=1.45, 95%CI: 1.02–2.06) and AA+AT vs. TT (OR=1.27, 95%CI: 1.06–1.50) genetic models. However, for the SNPs at rs8050136 and rs1421085, significant association was only found under AA vs. AC+CC (OR=1.65, 95%CI: 1.03–2.64, Fig. 3) and CC vs. CT+TT (OR=1.62, 95%CI: 1.08–2.42, Fig. 4), respectively.

Egger’s test (Table 3) showed no publication bias in any genetic model of FTO gene (*P*>0.05), which indicated that the pooled results in this meta-analysis were credible.

**Table 3:** The Publication bias assessment of the pooled results of the genetic models

**Gene**	**Gene model**	**Egger’s test for publication bias**	
		***t***	***P***
rs9939609	A vs. T	1.1887	0.3003
	AT vs. TT	−1.5718	0.1911
	AA vs. TT	0.3726	0.7284
	AA vs. TT+AT	1.4325	0.2114
	AA+AT vs. TT	−1.3029	0.2626
rs8050136	A vs. C	1.3115	0.3200
	AC vs. CC	1.3872	0.2997
	AA vs. CC	−0.1548	0.9022
	AA vs. CC+AC	−0.0861	0.9453
	AA+AC vs. CC	1.752	0.2219
rs1421085	C vs. T	3.1323	0.0886
	CT vs. TT	−0.5850	0.6178
	CC vs. TT	0.8490	0.4853
	CC vs. TT+CT	0.4394	0.7033
	CC+CT vs. TT	−0.3402	0.7661

## Discussion

In this study, we conducted a meta-analysis on the association between FTO gene polymorphisms and the risk of PCOS. The mutation at rs9939609 loci of FTO could significantly increase the risk of PCOS. However, no reliable evidence can identify the relationship between the other two SNPs of FTO (rs8050136 and rs1421085) and PCOS.

Our results are opposite to a previous meta-analysis which states that rs9939609 variant of FTO gene has no association with PCOS (24). Rs9939609 is a common polymorphism of FTO gene and has been identified to link with obesity. PCOS patients had significantly higher mean BMI compared to the people without PCOS (25). However, most of the PCOS patients (81.3%) were not obese. Interestingly, the researchers (25) found that rs9939609 was not significantly associated with PCOS, but significantly associated with obese PCOS. Therefore, rs9939609 variant was not the major decisive factor of PCOS, and FTO might influence PCOS through an association with obesity. However, rs9939609 in FTO was associated with the risk of Chinese PCOS women both in obese and lean cases (19). The same result was found in rs1421085 and rs8050136 loci in Korean PCOS women after adjusting BMI (8). Thus, we guess that race might play a role in the association between FTO gene polymorphism and PCOS. Unfortunately, we did not adjust the obesity and race of the participants by meta-regression in this meta-analysis because of lack of the included studies and demographic characteristics of the included populations. This may be also a source of significant heterogeneity in pooled analysis. Nonetheless, it could remind us to consider these important factors when performing experiment design in future.

Except for obesity and race, metabolic syndrome may be another important factor influencing the association between FTO polymorphism and PCOS. Rs1421085 (C/T) polymorphism in FTO showed a significant association with obese PCOS women or PCOS patients with metabolic syndrome, but not associated with lean PCOS patients or controls (15). In addition, the type of PCOS should be also considered. In the 12 included studies, only one referred to the type of PCOS (17), and no association was found between FTO variation and insulin resistant phenotype of PCOS in that study. Moreover, it is very necessary to adjust more potential confounders to clarify the association between FTO polymorphism and PCOS.

There are several limitations in this meta-analysis. Firstly, some covariates were not adjusted because of lack of studies and incomplete data. Secondly, the influences of some variates of FTO (rs1121980, rs1558902, rs11642841, and rs17817449) on PCOS were not meta-analyzed due to insufficient data. Thirdly, HWE test showed disequilibrium in controls in several included studies. Therefore, these controls may not predict the condition of the population in a certain region. Despite all this, our study can provide an important reference for the further researchers and help them understand the association between FTO polymorphism and PCOS systematically.

## Conclusion

Rs9939609 SNP variation in FTO gene can significantly increase the risk of PCOS in women. However, rs8050136 and rs1421085 are associated with PCOS under recessive model, but no association under other genetic models. Therefore, the association between these two SNPs and PCOS are still unclear. Further studies with large-scale and high quality are needed to elucidate the association between them. Our study would provide reference for understanding the relationship between FTO and the risk of PCOS and help clinicians take strategy to treat PCOS.

## Ethical considerations

Ethical issues (Including plagiarism, informed consent, misconduct, data fabrication and/or falsification, double publication and/or submission, redundancy, etc.) have been completely observed by the authors.
